# Treatment of Lenalidomide Exposed or Refractory Multiple Myeloma: Network Meta-Analysis of Lenalidomide-Sparing Regimens

**DOI:** 10.3389/fonc.2021.643490

**Published:** 2021-04-14

**Authors:** Cirino Botta, Enrica Antonia Martino, Concetta Conticello, Francesco Mendicino, Ernesto Vigna, Alessandra Romano, Giuseppe Antonio Palumbo, Claudio Cerchione, Giovanni Martinelli, Fortunato Morabito, Francesco Di Raimondo, Massimo Gentile

**Affiliations:** ^1^ Hematology Unit, “Annunziata” Hospital of Cosenza, Cosenza, Italy; ^2^ Division of Hematology, AOU Policlinico, Catania, Italy; ^3^ Department of Medical and Surgical Sciences and Advanced Technologies “G.F. Ingrassia”, University of Catania, Catania, Italy; ^4^ Department of Medical Oncology, Istituto Scientifico Romagnolo per lo Studio e la Cura dei Tumori (IRST) IRCCS, Meldola, Italy; ^5^ Hematology and Bone Marrow Transplant Unit, Hemato-Oncology Department, Augusta Victoria Hospital, East Jerusalem, Israel

**Keywords:** myeloma, network meta analysis, daratumumab, lenalidomide, bortezomib, carfilzomib, isatuximab

## Introduction

Over the past 10 years, the treatment of multiple myeloma (MM) dramatically changed due to the introduction of a number of new agents and combination regimens both in the frontline and in the relapsed/refractory setting. Currently, at least 11 classes of therapeutic agents, including steroids, alkylators (melphalan and cyclophosphamide), proteasome inhibitors (PI: bortezomib, carfilzomib, ixazomib), immunomodulatory agents (thalidomide, lenalidomide, pomalidomide), monoclonal antibodies (mAbs: elotuzumab, daratumumab), HDAC-inhibitors (panobinostat), BCL2 inhibitors (venetoclax), selective inhibitors of nuclear export (selinexor), drug-conjugated mAbs (belantamab mafodotin), bispecific agents and CAR-T, are approved (or are going to be approved) alone or in different combinations for the treatment of this disease, while few or no data are available to guide the therapeutic strategy to adopt at diagnosis or relapse (1). The choice of the treatment at relapse (2), in particular, poses particular challenges, and is currently dependent on patients (age, comorbidities, fitness, renal impairment, frailty) and disease characteristics (aggressive vs biochemical relapse, cytogenetics, presence of extra-medullary disease), previous treatments (classes of agents, duration of response, progression while on therapy), regional drug access (approval of combinations, reimbursement, costs) and, finally, patient’s choice. Unfortunately, there is a lack of trials specifically designed to help in this choice, and often, pre-planned subgroup analyses, do not include a sufficient number of patients to reach statistical evidence. Recently, since lenalidomide is progressively becoming the preferred one-line option to treat MM patients (and often, it is administered until progression), the choice of the treatment to be offered at relapse should be carefully evaluated. Interestingly, it has been reported that the longest prior lenalidomide treatment duration (>12 months) and IMiD-free interval (>18 months) could positively impact patients’ outcome (3), making the choice of a lenalidomide-sparing regimen of particular interest in this setting. On the bases of these premises, we performed a systematic review and a frequentist network meta-analysis in R [by using the *netmeta* package (4)] comparing direct and indirect evidence on the efficacy of seven different lenalidomide-sparing regimens (bortezomib-dexamethasone, VD; daratumumab-VD, DVD; carfilzomib-D, KD; daratumumab-KD, KdD; pomalidomide-VD, PVD; isatuximab-KD, IKD; selinexor-VD, SVD) in lenalidomide-exposed and lenalidomide-refractory patients, to provide statistical evidence to support clinical decision making (**Supplementary Figure 1**).

## Evidence from Clinical Trials

**Figure 1:**
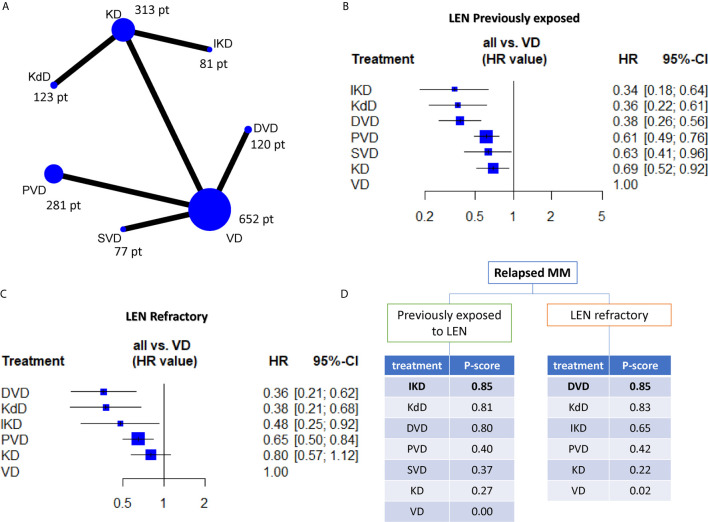
**(A)** Network plot showing all the direct comparisons and the number of patients included in each node (i.e. the total number of patients receiving the treatment indicated in the node). **(B, C)** forest-plots indicating the efficacy of each regimen (in terms of hazards ratio (HR) and 95% confidence intervals) by using VD as comparator arms. **(D)** Ranking charts of all the evaluated regimens based on the P-score and grouped according to previous exposition or resistance to lenalidomide. VD, bortezomib-dexamethasone; DVD, daratumumab-VD; SVD, selinexor-VD; PVD, pomalidomide-VD; KD, carfilzomib-dexamethasone; KdD, daratumumab-KD; IKD, isatuximab-KD.

Overall, we included 1,616 relapsed refractory MM patients (RR/MM) previously exposed to lenalidomide (lena-exposed) and 984 RR/MM patients reported to be lenalidomide refractory (lena-refractory) included in six randomized phase 3 trials (5–10). **Figure 1A** (and **Supplementary Figure 1**) reports the distribution of patients according to treatment and the presence of direct comparisons. All the groups were well balanced for presence of lena-refractory patients (about 70%, with the exception of the Castor trial which, within the lena-exposed population, only included about 50% of lena-refractory patients), exposure to bortezomib (about 65%, with the exception of the aCD38_KD group were about 90% of patients have been previously exposed to bortezomib) (**Table 1**) and patients treated in second line (about 45% in all trials, data not shown). Hazard ratios for PFS were included in our study. As reported in **Figures 1B** and **C**, all the treatments appear to be significantly superior to VD in both the lenalidomide exposed and refractory setting (with the exception of KD in the refractory group). Interestingly, DVD resulted to be significantly better than VD, KD, and PVD, slightly better than SVD (without reaching the statistical significance) and equal to both IKD and KdD in the lena-exposed population (**Supplementary Figure 2**). The same results are observed within the lena-refractory population, where DVD shows a trend of superiority over PVD and a significant advantage over both KD and VD. Looking at the P scores (the equivalent of the SUCRA score in frequentist NMA (4)), the triplets including an anti-CD38 mAb and a PI, always outperforms PVD and the doublets VD and KD (**Figure 1D**). These results are in line with our previous work where we demonstrated in pairwise meta-analysis the advantage of triplets over doublets in the RRMM setting (1).

**Table 1:** Main characteristics and previous treatments of the clinical trials included in the network meta-analysis.

Trials/Authors	treatment	total pts	LEN previously exposed	LEN refractory	%	BORT previously exposed	%
Castor/Palumbo	DVD	251	89	45	50,6	162	64,5
	VD	247	120	60	50,0	164	66,4
Endeavor/Dimopoulos	KD	464	177	113	63,8	250	53,9
	VD	465	177	122	68,9	252	54,2
Optimismm/Richardson	PVD	281	281	200	71,2	201	71,5
	VD	278	278	191	68,7	203	73,0
Candor/Dimopoulos	KdD	312	123	99	80,5	287	92,0
	KD	154	74	55	74,3	134	87,0
Ikema/Moreau	IKD	179	81	57	70,4	166	92,7
	KD	123	62	42	67,7	105	85,4
Boston/Dimopoulos	SVD	195	77			134	68,7
	VD	207	77			145	70,0

VD, bortezomib-dexamethasone; DVD, daratumumab-VD; SVD, selinexor-VD; PVD, pomalidomide-VD; KD, carfilzomib-dexamethasone; KdD, daratumumab-KD; IKD, isatuximab-KD.

## Discussion

Currently, no guidelines exist, which help decision making in the lenalidomide exposed or refractory setting. The last European guideline (11), indicates, due to the plethora of new agents currently available for MM treatment, to perform a class-switch whenever possible at the time of relapse, without indicating the best regimen to choose. The absence of precise indications mainly depends on the lack of direct comparisons between the available regimens together with the lack of preplanned subgroup analysis “numerically” designed to answer these questions. To overcome this limitation, we used the NMA approach, demonstrating that, whenever possible, the combination of an anti-CD38 agent with a PI should represent the first choice in order to achieve the best result in term of PFS in both the lena-exposed and lena-refractory RRMM population. However, while these results are of strong clinical interest, some limitations due to the methodology should be taken into account: for instance, the NMA is based on indirect evidence (other than on direct evidence), which could intrinsically introduce biases and on data retrieved from published studies rather than from individual patients. Additionally, while patients’ characteristics are very similar between the studies included in the NMA, small or unknown differences, such as the distribution of patients according to treatment line, could impact the final results: e.g., the CASTOR trial (10) includes, within the lena-exposed group, about 50% of patients refractory to lenalidomide, which is a lower than what reported in the other trials; however the advantage of DVD was confirmed even in the lena-refractory subgroup, rendering this difference acceptable. Furthermore, few or no data are currently available on the activity of lenalidomide-based triplets or quadruplets (thus excluded from this analysis) in lena-refractory patients as well as on the efficacy of lenalidomide ramp-up in patients progressing during 10 mg maintenance. Along the same line, the efficacy of the new pomalidomide/mAbs combo regimens, which look very promising, could not be evaluated with this approach, mainly due to the fact that all the investigational clinical trials have been performed in more advanced settings (from the third line of therapy) by using (always) PD as control arm (12–14). Translational investigations, which shed light on the biologic interplay which take place within the bone marrow microenvironment (15–19) are eagerly awaited and could help to develop new therapeutic approaches in this setting. Finally, this work should be considered a snapshot of current evidence, taking into account that some of these drugs will probably move to the frontline setting.

To the best of our knowledge, this is the first NMA designed to compare the efficacy of lena-sparing regimens in RRMM previously exposed or refractory to lenalidomide. Our findings suggest that among the currently approved regimens, DVD (or KdD/IKD when available) has the highest probability of being the best treatment in both lenalidomide previously exposed or refractory setting, further underscoring how mAbs represents a very important addition to the therapeutic armamentarium available for the treatment of MM patients. However, taking into account that, even with these regimens, the reported median PFS is about 9 months, prospective randomized trials investigating new agents and combinations are needed to identify better therapeutic options for this high-risk MM population.

## Author Contributions

CB designed the research and analyzed data. MG and FMe supervised the analysis. EM, CCo, CCe, and AR provided analysis and discussion inputs. CB and MG wrote the paper. FD, EV, FMe, GM, GP, and FMo revised the paper and improved the discussion of the results. All authors contributed to the article and approved the submitted version.

## Conflict of Interest

The authors declare that the research was conducted in the absence of any commercial or financial relationships that could be construed as a potential conflict of interest.

## References

[1] Botta C Ciliberto D Rossi M Staropoli N Cuce M Galeano T. Network meta-analysis of randomized trials in multiple myeloma: efficacy and safety in relapsed/refractory patients. Blood Adv (2017) 1:455–66. 10.1182/bloodadvances.2016003905 PMC5738982 29296961

[2] Offidani M Boccadoro M Di Raimondo F Petrucci MT Tosi P Cavo M. Expert Panel Consensus Statement for Proper Evaluation of First Relapse in Multiple Myeloma. Curr Hematol Malig Rep (2019) 14:187–96. 10.1007/s11899-019-00507-x 31077067

[3] Kastritis E Roussou M Gavriatopoulou M Kanellias N Migkou M Eleutherakis-Papaiakovou E. Impact of last lenalidomide dose, duration, and IMiD-free interval in patients with myeloma treated with pomalidomide/dexamethasone. Blood Adv (2019) 3:4095–103. 10.1182/bloodadvances.2019000539 PMC6963239 31821457

[4] Rucker G Schwarzer G. Ranking treatments in frequentist network meta-analysis works without resampling methods. BMC Med Res Methodol (2015) 15:58. 10.1186/s12874-015-0060-8 26227148 PMC4521472

[5] Dimopoulos M Quach H Mateos MV Landgren O Leleu X Siegel D. Carfilzomib, dexamethasone, and daratumumab versus carfilzomib and dexamethasone for patients with relapsed or refractory multiple myeloma (CANDOR): results from a randomised, multicentre, open-label, phase 3 study. Lancet (2020) 396:186–97. 10.1016/S0140-6736(20)30734-0 32682484

[6] Dimopoulos M Weisel K Moreau P Anderson LD Jr. White D San-Miguel J. Pomalidomide, bortezomib, and dexamethasone for multiple myeloma previously treated with lenalidomide (OPTIMISMM): outcomes by prior treatment at first relapse. Leukemia (2020). 10.1038/s41375-020-01021-3 PMC8179841 32895455

[7] Dimopoulos MA Delimpasi S Simonova M Spicka I Pour L Kryachok I. Weekly selinexor, bortezomib, and dexamethasone (SVd) versus twice weekly bortezomib and dexamethasone (Vd) in patients with multiple myeloma (MM) after one to three prior therapies: Initial results of the phase III BOSTON study. J Clin Oncol (2020) 38:8501–1. 10.1200/JCO.2020.38.15_suppl.8501

[8] Dimopoulos MA Goldschmidt H Niesvizky R Joshua D Chng WJ Oriol A. Carfilzomib or bortezomib in relapsed or refractory multiple myeloma (ENDEAVOR): an interim overall survival analysis of an open-label, randomised, phase 3 trial. Lancet Oncol (2017) 18:1327–37. 10.1016/S1470-2045(17)30578-8 28843768

[9] Martin TG Dimopoulos MA Yong K Mikhael J Risse M-L Asset G. (IKEMA) study design: Isatuximab plus carfilzomib and dexamethasone (Kd) vs Kd in patients with relapsed/refractory multiple myeloma (RRMM). J Clin Oncol (2018) 36:TPS8060–TPS8060. 10.1200/JCO.2018.36.15_suppl.TPS8060

[10] Palumbo A Chanan-Khan A Weisel K Nooka AK Masszi T Beksac M. Daratumumab, Bortezomib, and Dexamethasone for Multiple Myeloma. N Engl J Med (2016) 375:754–66. 10.1056/NEJMoa1606038 27557302

[11] Moreau P San Miguel J Sonneveld P Mateos MV Zamagni E Avet-Loiseau H. Multiple myeloma: ESMO Clinical Practice Guidelines for diagnosis, treatment and follow-up. Ann Oncol (2017) 28:iv52–61. 10.1093/annonc/mdx096 28453614

[12] Attal M Richardson PG Rajkumar SV San-Miguel J Beksac M Spicka I. Isatuximab plus pomalidomide and low-dose dexamethasone versus pomalidomide and low-dose dexamethasone in patients with relapsed and refractory multiple myeloma (ICARIA-MM): a randomised, multicentre, open-label, phase 3 study. Lancet (2019) 394:2096–107. 10.1097/01.HS9.0000561576.58696.ae 31735560

[13] Siegel DS Schiller GJ Samaras C Sebag M Berdeja J Ganguly S. Pomalidomide, dexamethasone, and daratumumab in relapsed refractory multiple myeloma after lenalidomide treatment. Leukemia (2020) 34:3286–97. 10.1038/s41375-020-0813-1 PMC7685974 32376855

[14] Dimopoulos MA Dytfeld D Grosicki S Moreau P Takezako N Hori M. Elotuzumab plus Pomalidomide and Dexamethasone for Multiple Myeloma. N Engl J Med (2018) 379:1811–22. 10.1056/NEJMoa1805762 30403938

[15] Botta C Cuce M Pitari MR Caracciolo D Gulla A Morelli E. MiR-29b antagonizes the pro-inflammatory tumor-promoting activity of multiple myeloma-educated dendritic cells. Leukemia (2018) 32:1003–15. 10.1038/leu.2017.336 PMC5886056 29158557

[16] Botta C Di Martino MT Ciliberto D Cuce M Correale P Rossi M. A gene expression inflammatory signature specifically predicts multiple myeloma evolution and patients survival. Blood Cancer J (2016) 6:e511. 10.1038/bcj.2016.118 27983725 PMC5223153

[17] Cuce M Gallo Cantafio ME Siciliano MA Riillo C Caracciolo D Scionti F. Trabectedin triggers direct and NK-mediated cytotoxicity in multiple myeloma. J Hematol Oncol (2019) 12:32. 10.1186/s13045-019-0714-9 30898137 PMC6429746

[18] Perez C Botta C Zabaleta A Puig N Cedena MT Goicoechea I. Immunogenomic identification and characterization of granulocytic myeloid-derived suppressor cells in multiple myeloma. Blood (2020) 136:199–209. 10.1182/blood.2019004537 32325491

[19] Rossi M Altomare E Botta C Gallo Cantafio ME Sarvide S Caracciolo D. miR-21 antagonism abrogates Th17 tumor promoting functions in multiple myeloma. Leukemia (2020) 136(2):199–209. 10.1038/s41375-020-0947-1 32632096

