# Supplementary figures and images for: Treatment of Lenalidomide Exposed or Refractory Multiple Myeloma: Network Meta-Analysis of Lenalidomide-Sparing Regimens
Source: Front Oncol. 2021 Apr 14;11:643490. doi: 10.3389/fonc.2021.643490 (PMC8079718; doi:10.3389/fonc.2021.643490)

Supplementary Fig. 1

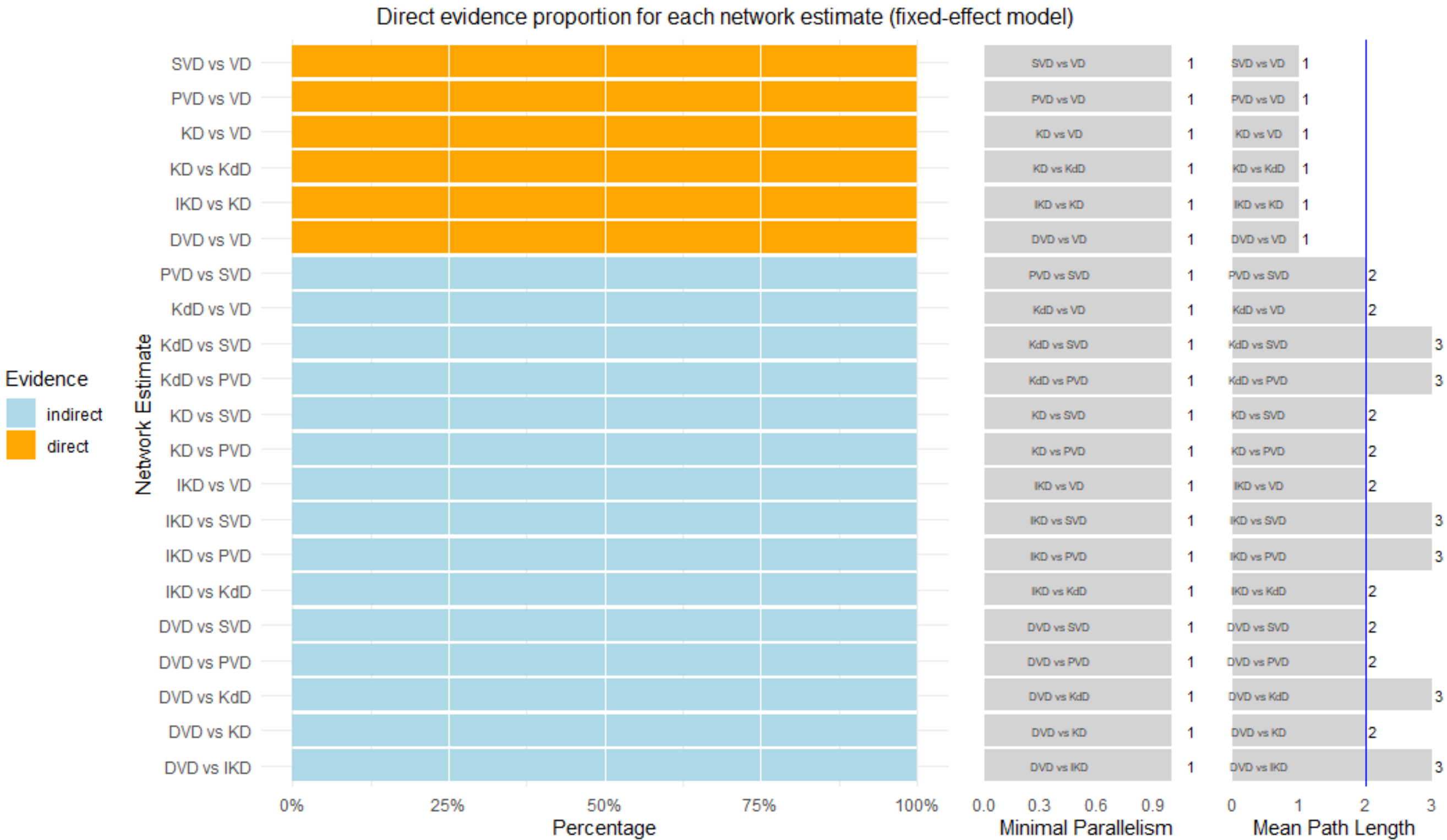

Supplementary Fig. 2

LEN Previously exposed

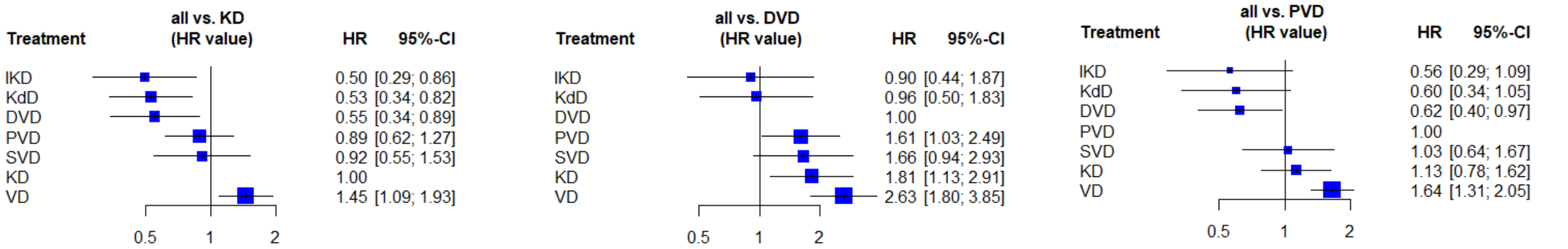

LEN refractory

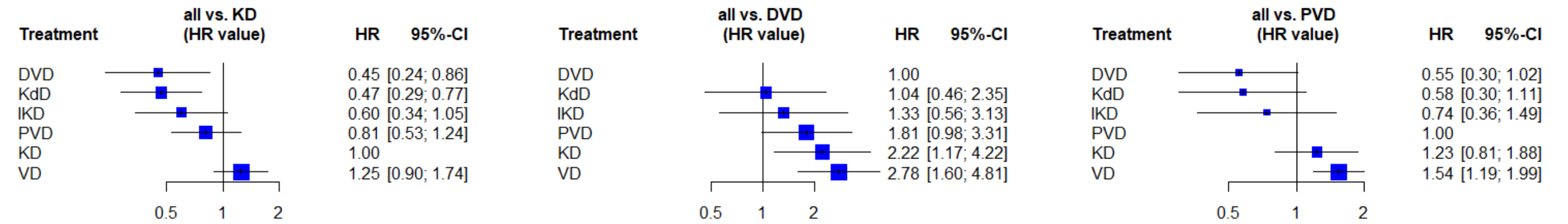

Supplement: Supplementary file 1 [file DataSheet_1.pdf]
